# Sense of Coherence, Burnout, and Work Engagement: The Moderating Effect of Coping in the Democratic Republic of Congo

**DOI:** 10.3390/ijerph17114127

**Published:** 2020-06-10

**Authors:** Jeremy Mitonga-Monga, Claude-Hélène Mayer

**Affiliations:** Department of Industrial Psychology & People Management, University of Johannesburg, Johannesburg 2006, South Africa; jeremym@uj.ac.za

**Keywords:** sense of coherence, employee participation, burnout, work engagement, Democratic Republic of Congo (DRC)

## Abstract

Research on coping, sense of coherence, burnout, and work engagement is well documented in western countries. However, a void of studies exists on how coping mechanisms can moderate the relationship among sense of coherence, burnout, and work engagement in a manufacturing company in the Democratic Republic of Congo (DRC). The objective of this research was to examine the moderating effect of coping (COP) in the relationship between sense of coherence (SOC), burnout (BO), and work engagement (WE). The study employed a quantitative research approach, while participants were recruited through convenience sampling. A total of 197 employees (*n* = 197; females 40%) who are permanently employed in a manufacturing organisation in the DRC participated in the study voluntarily. The results indicate that coping related positively to a sense of coherence. Moreover, the results indicate that sense of coherence and work engagement related negatively to burnout. Furthermore, the results show that coping acted as a moderator in the relationships between variables. The study adds value to the WE theory by suggesting that an employee who has a high level of COP, high SOC, low level of BO, will positively engage, perform, and be productive.

## 1. Introduction

Work engagement (WE) is a topic of popular interest in the field of management and industrial and organisational psychology, internationally, as well as in Sub-Saharan African contexts [1,2,3]. WE is described as a positive, fulfilling work-related state of mind that is characterised by rigor, dedication, and absorption [4]. Previous research on WE reveals that WE decreases levels of occupational stress [5,6] and brings about organisational and financial success [7]. Work engagement refers to an energetic state in which the employees are devoted to excellent performance at work, whilst being confident about their own effectiveness [8]. It relates to work-related outcomes, promoting employee health and well-being [9], productivity and flexibility [10], individual morale, and extra-role and organisational performance [11]. Previous research established that a high-level sense of coherence (SOC) influences employees’ perceptions of their leader’s behaviour [12], work engagement [13], coping strategies [14], and relate negatively to burnout [15].

This research study probes the moderating effect of coping in the relationship between sense of coherence (SOC), burnout (BO), and work engagement (WE). This article investigates the above-mentioned relationships of COP, SOC, BO, and WE in a developing country setting, particularly in the Democratic Republic of Congo (DRC), where organisations remain comparably ineffective, and need to learn how to cope with the demands of the changing work environment in the context of WE. This study, therefore, investigates, how employees of a manufacturing organisation in the DRC perceive the COP, SOC, and BO to affect their level of WE.

## 2. The Democratic Republic of Congo’s Work Context

The DRC is a developing country, with considerable economic potential because of its vast mineral and natural wealth [16]. However, political and economic instability have resulted in high levels of inflation, unemployment, and liquidation of companies, retrenchments, corruption, and the under-development of infrastructure [17]. Security and human rights within workplaces are limited [18], which has resulted in the country ranked below 7% on all six indicators [12], with the lowest scores on government effectiveness, rule of law, political stability, and control of corruption [16]. The country’s manufacturing sector lacks basic infrastructure, while employees perceive poor leadership, which impacts production negatively. A previous study by Mitonga-Monga, Coetzee and Cilliers [19] found that EP is predicted by leadership style, SOC, WE and BO. In their study on BO in the DRC, Wolf, Torrente, McCoy, Rasheed and Aber [20] reported that years of experience would influence the association between BO and cumulative risk. Literature and previous research on how COP influences the association between SOC, BO, and WE in the DRC is limited; therefore, hardly any previous research that was done in this context can be presented here. This study aims to fill the void of organisational research in the DRC.

## 3. Theoretical Background

### 3.1. Salutogenesis and Sense of Coherence (SOC)

Salutogenesis is the science of the development of health [21]. It is based on the SOC, which refers to a global life orientation that expresses the extent to which one has a pervasive, enduring, dynamic feeling of confidence, that one’s internal and external environments are structured, both predictably and explicably, and that there is a high probability that tasks can be managed, and are worth managing [21,22,23].

The three SOC components are defined as follows [21,24,25]: (1) comprehensibility—this refers to the extent to which individuals find or structure their world in order to be understandable, meaningful, orderly and consistent instead of chaotic, random and unpredictable; (2) manageability—this refers to the extent to which individuals experience events in life as situations that are endurable or manageable and, which can even be seen as new challenges; and (3) meaningfulness—this refers to the extent to which one feels that life makes sense on an emotional level and not merely on a cognitive level, and that life’s demands are worthy of commitment.

Research on SOC [14,21,22,26,27,28] has shown this construct to be an important component of individuals’ health and well-being. It acts as an effective coping strategy [4,29]. It manifests as a readiness and willingness to utilise resources at their disposal [30] to appraise, understand, and make sense of their complex reality and environment, and to choose appropriate strategies to deal with stressors and anxiety in spite of the adversity [31]. Previous research established that a higher level of COP, a strong SOC, and a low level of BO predict WE and performance [1,4].

For the DRC context, research shows that a high SOC relates positively to high levels of education, high income, and positive social relationships, and inversely correlates with cumulative exposure to violence, depression and PTSD symptoms [32]. Mitonga-Monga and Hlongwane [13] found in subsequent research on SOC in a manufacturing company that high levels of SOC perceptions influenced the relationship between leadership style and WE.

### 3.2. Burnout (BO)

BO refers to a persistent, negative work-related state of mind (or syndrome), which is characterised by an array of physical, psychological, and attitudinal symptoms [8,33]. It is a chronic, negative, affective response, with fatigue and emotional exhaustion as major symptoms [34].

The three dimensions of BO are defined as follows [35]: (1) exhaustion refers to the depletion or draining of emotional resources and feelings of being overextended, whilst experiencing distress, a sense of reduced effectiveness, decreased motivation and the development of dysfunctional attitudes and behaviours at work; (2) cynicism refers to interpersonal behaviour that manifests as a negative, callous or excessively detached response to various aspects of the job; and (3) professional efficacy refers to self-evaluation behaviour, which manifests as a feeling of competence, productivity, and achievement at work.

BO develops gradually among individuals who experience crises in their relationships with work, but not necessarily in their relationships at work [33]. It manifests as a persistent, negative, work-related state of mind, which is mediated by self-efficacy beliefs and emotional stability [36]. Researchers have indicated that the development of BO is also characterised by a lack of proper promotion possibilities, policy, and the inability of employees to achieve career goals [19]. Research in the DRC shows that the experience of higher job risks relates to lower motivation and higher BO levels and, therefore, decreased mental well-being [37]. Research in the DRC shows that the experience of higher job risks relates to lower motivation and higher BO levels and, therefore, decreased mental well-being [20]. However, research on BO in industrial work-related contexts are hardly to be found in the DRC. Research in the DRC shows that the experience of higher job risks relates to lower motivation and higher BO levels and, therefore, decreased mental well-being [20]. However, research on BO in industrial work-related contexts are hardly to be found in the DRC. Previous research by Mitonga-Monga, Coetzee, and Cilliers [19] reported that BO related negatively to SOC, EP, and WE.

### 3.3. Work Engagement (WE)

WE refers to a positive, fulfilling work-related state of mind [9,38]. Rothman et al. [27] indicate that it is not momentary or specific, but rather a more persistent and pervasive affective-cognitive state that is not focused on a particular object, event, individual, or behaviour. Employees who are strongly engaged in their daily work display intrinsic motivation through dedication to their jobs and are described as persistent and involved in their work [39]. WE has been frequently linked to work-related outcomes, including health and well-being, productivity and reduced turnover, and stress [40]. The three dimensions of WE include [10,41,42]: (1) vigour–refers to high levels of energy and mental resilience while working, as well as a willingness to exert effort and perseverance even during difficult times; (2) dedication–refers to a sense of significance in terms of one’s work, feeling enthusiastic, inspired and proud, and viewing one’s job as a challenge; and (3) absorption–refers to a satisfactory state of complete emersion in one’s work, as well as focused attention, time distortion, loss of self-consciousness, effortless concentration, absolute control and intrinsic enjoyment. WE relates reciprocally to self-efficacy, positive affect and enthusiasm at work [15]. Work engaged employees with a strong SOC are further likely to be involved in decision-making processes and exhibit low levels of BO [40]. Previous study by Mitonga-Monga and Hlongwane [13] indicates that work engaged employees display intrinsic motivation through dedication to their jobs, work persistence, while they focus on their task performance.

### 3.4. Coping Strategies

Coping strategies have been increasingly researched by scientific scholar during the past decade as important resources in challenging life situations [43]. To comprehend how people positively face adversity is crucial and important to know the factors that may contribute to or promote resilience [44]. Coping has been described as a person’s efforts to alleviate, reduce, or manage menacing events that are appraised as challenging or stressful [45]. Prior research endeavours have clustered coping mechanism into two factors, namely problem-focused and emotion-focused [45]. The latter, is aimed at regulating distress and negative emotion rather than trying to change the events themselves, using strategies such as escape support seeking or avoidance. Problem-focused comprises addressing the problem causing the distress the effective problem-focused contribute to positive psychological state by permitting individuals to experience some personal control and a sense of achievement [46].

The literature on coping has further distinguished between active and avoidant coping strategies [47]. Active coping strategies are perceived as behavioural or psychological reactions intended to change the nature of the stressor themselves or how one thinks about them. Whereas, avoidant coping strategies lead individuals into activities, such as alcohol use or destructive mental states, such as withdrawal that prevent them from addressing the stressor directly [47].

Previous research has found that individual who cope with stress by seeking social support or voicing their feeling (emotion-focused) are likely to experience negative outcomes than individual who address the experienced stressor immediately by working on find solutions to their problems [47]. A study by Pisula and Kossakowska [48] on SOC and COP in a sample of mothers and fathers of children with autism, found that a high level of SOC related positively to seeking social support and self-controlling and negatively with accepting responsibility. A previous study by Rothmann and Jorgensen [49] found that WE related positively to problem-focused coping, positive reinterpretation, and growth. High level of COP was found to play an important role in dealing with occupational stress [50]. The following section discusses inter-linkages of the four constructs described above.

### 3.5. Sense of Coherence, Burnout and Work Engagement Relationships

Researchers are investigating the link between COP, SOC, BO, and WE [3,26,49]. Several studies demonstrate that SOC relates positively to employees’ level of WE, work involvement and stress [20], and influences the ability to mobilise and generate social resources in the workplace [51]. SOC predicts WE in different cultural groups in South Africa [3]. A strong SOC positively relates to WE, and negatively to the exhaustion and cynicism dimensions of BO [27]. Employees with a weak SOC and a low level of WE tend to develop BO and be less involved and engaged in their work. May, Gilson, and Harter [50] point out that WE relates particularly positively to meaningfulness, which is the third component of SOC. Employees who have developed a strong SOC show WE, are more involved, and actively participate in their work [1,4,20]. WE and BO are conceptualised to be opposing constructs on the ease-disease continuum, while vigour and dedication are the direct opposites of exhaustion and mental distance (cynicism) [3]. Previous research has found that WE and BO relate closely to work-associated well-being [8,52], while managing job burnout prevents ill-health outcomes [1,41,53].

Research has established that BO relates negatively to WE, as it does to SOC [8]. In order to facilitate WE and prevent BO, organisational contexts should foster environments where employees feel enthusiastic, energised, and motivated [54]. However, WE does not always lead to high performance, nor does high performance always indicate WE [55]. This means that employees may show initiative and take responsibility; not because they feel engaged, but rather because they fear redundancy and want to prove their capability. Conversely, employees might fail to show initiative; not because they are unengaged, but rather because constraints in the environment inhibit them from displaying their initiative [55].

### 3.6. COP as Moderator

Several empirical studies have examined the influence of COP in the association between occupational stress, SOC, BO, and WE [10,48], stress mindset, and psychological stress response [56]. However, the findings of these studies were divergent. For example, Rothmann, Jorgensen and Marais [49] reported that high problem-focused, seeking social support, turning to religion and low ventilation of emotions predicted work engagement. Thus, Rodrigues et al. [57] found that coping and stress appraisals do not seem to predict work engagement. Although some authors argue that COP and SOC constructs are likely to reduce individual level of stress [44]. Furthermore, Basson and Rothmann [58] found that both SOC and COP predicted emotional exhaustion, depersonalisation, and personal accomplishment (BO). A study by Van der Colff and Rothmann [4] reported that SOC and ventilation of emotion, low seeking emotional/social support coping predicted emotional exhaustion. Individuals with a strong SOC, high level of confronting COP low burnout are likely to demonstrate a higher level of work engagement [58]. Pisula and Kossakowska [48] studied the relationship between SOC and coping with stress and they found that SOC related negatively to COP. Although a great deal has been learned about the association between SOC, BO, and WE in Western countries [59], little has been learned in African contexts on how COP may moderate the relationship among these variables [3,26,35]. This void is addressed here for the DRC context.

## 4. Purpose and Aim of the Study

The purpose of this study is to examine how COP moderate the relationships between SOC, BO, and WE in a manufacturing organisation in the DRC. This purpose is informed by the void in research, exploring inter-linkages of the four constructs in African contexts, particularly in the DRC. The results of this study contribute to the body of knowledge of COP, SOC, BO, and WE, and can be used to increase organisational health and well-being, WE, whilst decreasing BO. The study addresses the void of research in Industrial and Organisational Psychology within developing countries in Central Africa, and particularly in the DRC.

The following research question guides the investigation and the presentation of the results:

- How do employees’ levels of COP influence their level of SOC and BO?
- How do employees’ levels of COP influence their level of SOC and WE?

## 5. Research Methodology

### 5.1. Research Paradigm and Design

This study is framed in the positivist paradigm [60] to achieve objective truths, facts, and laws by using a quantitative methodology. It makes use of a non-experimental, quantitative approach, comprising a range of different methods, which aim to describe the relationship between constructs by testing any causal relationships between them [60].

### 5.2. Sample and Setting

A convenience sample of employees (*n* = 197; females = 40%) in a manufacturing company in the DRC was used (see Table 1 for the demographics). Table 1 indicates that participants were predominantly males (60%) who have a university degree education (61%) and are in their establishment career age (40–55 years). The minority of the participants were proportionally working in human resources, sales, technical, project management, and exploitation management (17%).

### 5.3. Measures

Four standardised questionnaires were used, namely one each for the a.m. constructs, as well as a biographical survey.

Coping Strategies Scale (CSC) [61] consists of 30 items self-reported instrument, measuring problem-focused and seeking support. It is scored on a four-point Likert-type-scale (1 = never, 4 = always). Examples of the items includes the following: problem-focused (tried to solve the problem); and seeking support (went to a friend for advice on how to change the situation), avoidance (Avoided being with people in general). This study obtained a Cronbach alpha coefficient of 0.65 for problem-focused COP, and 0.66 for seeking support COP (See Table 2).

The Sense of Coherence (SOC) [30] was used to measure the sense of coherence. The SOC consists of 29 items, using a seven-point Likert-scale ranging from 1 (very often) to 7 (very seldom or never). Example of the items included the following: comprehensibility (do you have the feeling that you are in an unfamiliar situation and don’t know what to do?); manageability (has it happened that people whom you counted on disappointed you); and meaningfulness (until now your life has had: no clear goals or purpose at all- very clear goals and purpose). This study obtained a Cronbach alpha coefficient of 0.61 for comprehensibility, and 0.78 for SOC (see Table 2).

The Maslash Burnout Inventory General Survey (MBI-GS) [62] consists of 16 items in the self-report instrument, which measured cynicism, exhaustion, and professional efficacy. It is scored on a seven-point Likert-type-scale (0 = never, 6 = every day). Examples of the items included the following: cynicism (I have become less enthusiastic about my work); exhaustion (I feel used up at the end of a working day); and professional efficacy (In my opinion, I am good at my job. This study obtained a Cronbach alpha coefficient of 0.61 for exhaustion, and 0.64 for cynicism, and 0.71 for total burnout BO (See Table 2).

The Utrecht Work Engagement Scale (UWES) [10] consists of 17 items in the self-report instrument, measuring vigour, dedication, and absorption. It is scored on a seven-point Likert- type-scale (0 = never, 6 = every day). Examples of the items included the following: vigour (I am bursting with energy in my work); dedication (I find my work full of meaning and purpose); and absorption (When I am working, I forget everything else around me. This study obtained a Cronbach alpha coefficient 0.64 for dedication, 0.77 for vigour, and 0.76 for total work engagement (WE) (See Table 2).

The researcher decided to exclude avoidance COP, manageability SOC and meaningfulness SOC, Exhaustion BO and professional efficacy BO, and absorption WE from the interpretation because of the low reliability.

### 5.4. Procedure

Permission to conduct the research was obtained from both the management of the manufacturing company involved in the study, as well as the Ethics Research Review Committee of the overseeing academic institution (No.11/40- AO22/SD-Form/2013). The research assistant distributed research packages amongst the participants, and these comprised of the following: the participant consent form; an invitation letter indicating the aim of the study; both the university and management’s approval letter; confirmation of the safekeeping and confidentiality of the responses; instructions on how to complete the instruments; and the actual three instruments, all in hard copy. On completion, each individual was requested to sign the consent form and include this with the completed instruments in an appropriate envelope. The envelope then had to be returned to the research assistant who, in turn, mailed it to the researcher.

### 5.5. Data Analysis

The researchers conducted the statistical analysis with the aid of SPSS program (SPSS Inc., Chicago, IL, USA) [52,63,64]. They investigated the multivariate outliers with Mahalonibis distance using the distribution function for Chi-square. After investigation, three cases did not satisfy the conditions of (*p* ≤ 0.01) (62). The three cases were considered to have presence of outliers; therefore, the researchers decided the exclude them from the analysis.

The researcher used the descriptive statistics to explore the data. They calculate the internal consistency of the measuring instruments using item analysis if Cronbach alpha deleted [65]. Because of the low reliability on avoidance COP, Manageability SOC and meaningfulness SOC, Exhaustion BO and professional efficacy BO and absorption WE sub-scales the researchers decided to exclude them from the interpretation [66]. The researchers used Pearson correlation coefficients to determine the relationships between the variables (Problem-focused, seeking support COP, comprehensibility SOC, vigour and dedication WE, and cynicism BO). The researchers used effect size [67] to determine the practical significance of the findings. They set a cut-off alpha value of 95% confidence interval level (*p* ≤ 0.05) and a practical effect size of r ≥ 0.11 (small effect size) to r ≥ 0.31 (medium effect size) were implemented.

The researchers conducted hierarchical multiple regression analyses to determine whether (1) COP moderate the relationship between sense of coherence and Burnout; (2) COP moderated the relationship between SOC and work engagement. The interactions were explored using a simple slope test and the value of the moderator at the −1SD mean +1SD, as well as standard deviations above and below the mean [66]. In order to counter the probability of type I errors, the significant value was set at the 95% confidence interval level (*p* ≤ 0.05). For the purpose of this study, the practical significance of *R^2^* values was determined by calculating effects sizes (*f*^2^) [68].

## 6. Results

### 6.1. Descriptive Statistics: Means and Standard Deviations

Table 2 presents descriptive statistics for the variables. As shown in Table 2, the participants obtained relatively high scores for the seeking COP (M = 3.13; SD = 0.49) and low scores on problem-focused COP (M = 2.69; SD = 0.55). In terms of the sense of coherence, participants obtained relatively high scores for comprehensibility SOC (M = 5.22; SD = 1.25), sense of coherence SOC (M = 4.64; SD = 0.72.)

As shown in Table 2 above, the participants obtained high scores burnout BO (M = 3.71; SD = 0.72), and cynicism BO (M = 2.20; SD = 1.66). In terms of work engagement, the participants obtained relatively high scores on vigour WE (M = 4.86; SD = 1.12), work engagement WE (M = 4.54; SD = 0.81) and dedication WE (M = 4.12; SD = 1.20).

### 6.2. Correlational Analysis

Table 3 also presents the significant correlation coefficients that were identified between the COP, SOC, BO, and WE variables. The inter-correlations ranged from r ≤ −0.14 (small practical effect size) to r ≥ 0.82 (large practical effect size). These results indicate that the zero-order correlations were below the threshold level of concern (r ≥ 0.90) of multi-collinearity. Problem-focused and seeking support COP positively related SOC and vigour WE and negatively related to and cynicism BO. SOC negatively and significantly related to BO variable. SOC positively and significantly related to vigour and absorption WE variables. BO related negatively and significantly to vigour and dedication WE (the *p* values ranged between *p* ≤ 0.001 and *p* ≤ 0.005).

### 6.3. Hierarchical Regression Analysis

Table 4 indicates the moderating effect results.

As indicated in Table 4 below, in terms of the main effects, total SOC did not act as a significant predictor of the BO. (F (3; 193) = 18.66; *p* ≤ 0.05), (B = −1.01; SE_B_ = 0.84; 95%CI = (−2.66; 0.64); *p* = 0.23), denoting that SOC was not associated with a decrease in the percentage of the BO. The interactions were explored using a simple slope test and by graphing the interactions using the value of the moderator at the mean, as well as standard deviations above and below the mean [66]. As shows in Table 4, COP did not act as a moderator in the relationship between SOC and BO. (F (3; 193) = 18.66; *p* ≤ 0.05), (B = 0.14; SE_B_ = 0.29; 95%CI = (−0.43; 0.70); *p* = 0.64).

**Figure 1:**
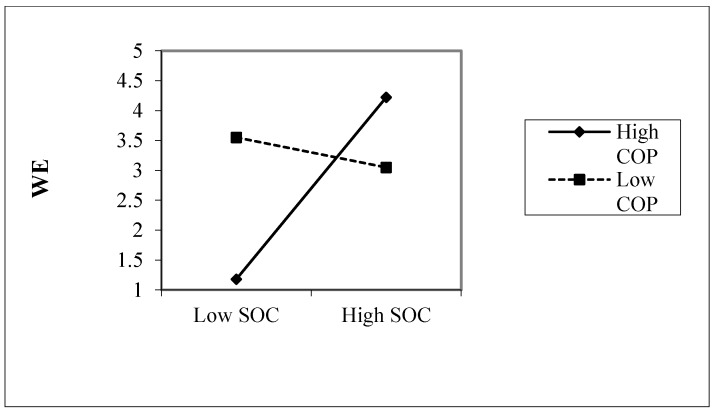
Interaction effect between COP, SOC, and work engagement (WE).

As indicated in Table 5 below, in terms of the main effects, SOC acted as a significant predictor of the WE. (F (3; 193) = 4.39; *p* ≤ 0.05), (B = 2.64; SE_B_ = 0.85; 95%CI = (0.96; 4.31); *p* < 0.05, denoting that SOC was associated with an increase in the percentage of the WE. Furthermore, COP acted as a significant predictor of the WE. (F (3; 193) = 4.39; *p* ≤ 0.05), (B = 4.30; SE_B_ = 1.34; 95%CI = (1.66; 9.94); *p* < 0.05), denoting that COP was associated with an increase in the percentage of the WE. The interactions were explored using a simple slope test and by graphing the interactions using the value of the moderator at the mean, as well as standard deviations above and below the mean [66] As illustrated in Figure 1, the relationship between SOC and WE was stronger for individuals with high level of COP than individual with low level of COP. The participants who scored high on COP also achieved significantly higher scores than their counterpart participants on the WE.

## 7. Discussion

Overall, the results suggest that participants’ perceptions of problem-focused COP relate significantly and positively to their perception of SOC and vigour WE. Moreover, their perceptions of problem-focused COP related negatively and significantly to their perceptions of cynicism BO. In addition, participants’ perceptions of seeking support COP related positively and significantly to their perception of SOC. Participants’ perceptions of SOC related significantly and negatively to their perceptions of cynicism BO. Furthermore, participant’s perceptions of SOC relate significantly and positively to their levels of vigour and dedication WE. These findings are consistent with those of prior research [23], which reported that a strong SOC and high problem-focused and high seeking support COP are important to foster the abilities and competences of employees to cope with diverse work-related challenges, whilst positively impacting work-related health and well-being. The results are likely to be explained by the fact that participants with a high SOC will likely reciprocate with a higher level of vigour WE [20].

The results suggest that problem-focused and seeking support COP related positively to SOC. This implies that individuals with a strong SOC and proper coping strategies are likely to overcome challenging events or stressful situations posed by their working environment. The results are likely to be explained by the fact that, participants with a strong SOC, who apply positive coping strategies, such as seeking support and problem-focus, are more likely to COP with stressful work environment. In contrast, participants with low level of SOC, are likely to experience threating situations, as they usually perceived stressors as a threat [43,44].

In addition, a low level of comprehensibility SOC relate to a higher level of BO. This implies that participants with a low comprehensibility SOC are likely to experience depletion of emotional resources, demonstrate cynical attitudes. In contrast, participants with a high comprehensibility SOC are less likely to have feelings of depletion of resources at work. These findings mirror those by Van der Colff and Rothmann [4] who found SOC to be negatively related with BO.

Further, the results suggest that high levels of BO relate to low levels of WE. This could possibly be explained by the fact that participants who have feelings of depletion, and who distance themselves emotionally and cognitively from their work are less likely to be energetic, enthusiastic, proud and engrossed in their work tasks. This study’s results support previous results for example, Van der Colff and Rothmann [4], which showed low level of WE related to higher levels of BO. These findings are consistent with those by Rožman et al. [8] who found WE to be negatively associate with BO. These results are particularly important in the context of the DRC, which aims to increase health and well-being amongst employees, whilst generally increasing performance in the manufacturing industry through promoting SOC, COP, WE and prevent BO. The present study revealed the important role of SOC in buffering BO, consistent with previous studies [4]; Participants with a strong SOC are likely to view a greater number of events as having coherence. This perceptual seem to be restrained: it influences individual’s perceptions of a stressful event, but it does so without their conscious awareness [4].

The results on the effects of COP on SOC and WE revealed that, participants with a strong SOC and high levels of WE perceived a high level of COP, then their counterparts with a low COP. This might be explained by the fact that when participants have higher level of COP, they might respond with high level of SOC and WE [4]. In other words, Participant with high-level of COP strategies and strong SOC are likely to cope with challenging situations and demonstrate high-level of energy, be enthusiastic, proud, and perform in their daily work. These findings mirror the ones of previous studies by Van Colff and Rothman [4], who found that participants with high COP and strong SOC are likely to seek emotional or social support when dealing with occupational stressors in a positive problem-focused manner. Participants with high level of COP and strong SOC are likely to demonstrate higher levels of WE, which in turn, will influence their well-being and ultimately, enhance its performance [10].

### 7.1. Limitations of the Study

The study comes with limitations. Conceptualisation of the study is limited by the fact that hardly any data is available concerning manifestation of positive psychology functioning in any middle, north, or West African country such as the DRC. This means that no comparisons with previous context-specific results could be conducted. In terms of psychometric procedures, translation of the instruments was potentially problematic in terms of experienced confusion about ideas and constructs from Western cultures implemented in culture-specific contexts such as the DRC. The sampling method (not being random) and low reliability prevented generalisation of the results from being applied beyond this organisation’s population.

### 7.2. Conclusions and Recommendations for Theory and Practice

The results suggest that employees become work-engaged and dedicated (high level of WE) when they perceive their world of work as being organised and structured. They demonstrate high levels of participation in their work, and are able to cope with their work, and see the meaning in their work (overall high SOC). These results are extremely important within the DRC-context since research regarding this context often focuses on negative aspects and pathogenetic approaches rather than on positive aspects and coping. This study therefore contributes to the positive psychology and positive organisational psychology literature with regard to the Central African context. If employees have a strong SOC, using positive coping strategies, and experiencing low levels of burnout, they would likely be work engaged. This study adds to the growing body of knowledge on COP, SOC, BO in the context of WE in Central African contexts and organisations and supports international studies on SOC and coping in generally challenging work situations. Results of this study support mainly Western-based literature and results from previous studies, however it might be assumed that the culture-specific motivations and the contextual influences and effects differ from Western research settings.

Research-related recommendations, therefore, include that Industrial and Organisational Psychologists should focus their research on constructs such as COP, SOC, BO, and WE in culture-specific contexts, compare them across countries (Pan-African research), and study them, particularly within African organisations in different sectors with mix-method approaches. Further, researchers should develop culture-specific quantitative research instruments to explore culture and language adequate concepts, and not only lean on Western research instruments. It is recommended that positive psychological functioning and its effect on work behaviour of employees and, particularly leadership, should be researched to further predict the way forward for employees and organisations in Central Africa into the fourth industrial revolution.

In terms of practical recommendations, Industrial and Organisational Psychologists and Human Resources Practitioners should become aware of the inter-linkages among COP, SOC, BO, and WE within this specific cultural and organisational context, and focus on positive psychological constructs and employee and organisational functioning, since in the past industrial research in African contexts focused mainly on problems and challenges. Programs that focus on the increase of SOC in terms of mental health and well-being within organisations should be developed with culture-specific backgrounds, particularly in a challenging context like the manufacturing sector in the DRC. The COP, SOC, and WE within organisations should be fostered to counteract BO and should contribute to an overall healthier and empowering work environment.

## Figures and Tables

**Table 1:** Sample profiles.

Characteristic	Category	Frequency	Percentage (%)
Gender	Male	118	60%
	Female	79	40%
Age	Less than 25 years	12	6%
	25–40 years	82	42%
	40–50 years	93	47%
	55 and older	10	5%
Education	Primary school	7	4%
	Secondary school	30	15%
	University bachelor & honours	118	60%
	Masters and doctorate	42	21%
Functional department	Human Resources	33	16%
	Financial management	33	17%
	Distribution & sales	33	17%
	Technical management	33	17%
	Project management	33	17%
	Exploitation management	32	16%

Source: Own data.

**Table 2:** Descriptive Statistics (Mean and Standard Deviation).

Variables	Mean	SD
Coping (COP)	2.90	0.31
Problem-Focused COP	2.69	0.55
Seeking support COP	3.13	0.49
Sense of coherence (SOC)	4.64	0.72
Comprehensibility (SOC)	5.22	1.25
Burnout (BO)	3.71	0.72
Cynicism (BO)	2.20	1.66
Work engagement (WE)	4.54	0.81
Vigour (WE)	4.86	1.12
Dedication (WE)	4.21	1.20

**Table 3:** Correlational analysis.

Variables	Copping COP	Problem-Focused COP	Seeking Support COP	Sense of Coherence SOC	Comprehensibility SOC	Burnout BO	Cynicism BO	Work Engagement WE	Vigour WE	Dedication WE
Coping (COP)	1	0.49 **	0.53 ***	0.70 ***	0.13	−0.15 *	−0.11	0.13	0.13	0.14
Problem-Focused (COP)		1	0.04	0.24 *	0.15	−0.15 *	−0.15 *	0.06	0.16*	−0.2
Seeking Support (COP)			1	0.11 *	0.03	0.03	0.13	0.03	0.02	0.05
Sense of Coherence (SOC)				1	0.73 ***	−0.47 **	−0.40	0.09	0.22 *	0.22 *
Comprehensibility (SOC)					1	−0.30 *	−0.28 **	−0.10	0.01	0.01
Burnout (BO)						1	0.82 ***	−0.25 *	−0.32 **	−0.36
Cynicism (BO)							1	−0.04	−0.14 *	−0.17 *
Work Engagement (WE)								1	0.82 ***	0.82 ***
Vigour (WE)									1	0.54 **
Dedication (WE)										1

*n* = 197; *** *p* ≤ 0.01, ** *p* ≤ 0.02, * *p* ≤ 0.05.

**Table 4:** Hayes’ Process Regression Matrix for Moderating effect of the coping (COP) on the relationship between sense of coherence (SOC) and (burnout (BO) (*n* = 197).

Variables	*B (SE_s_)*	*t*	*p*	95% Confidence Interval		*R*	*R^2^*
				LLCI	ULCI		
Constant	7.33 (3.80)	1.92	0.06	−0.18	14.84	0.47	0.22
SOC	−1.01 (0.84)	−1.20	0.23	−2.66	0.64		
COP	−0.64 (1.32)	−0.50	0.62	−3.25	1.94		
Interaction_1	0.14 (0.29)	0.47	0.64	−0.43	0.70		

Note: *B* = Unstandardized coefficients; SEs = standard errors; *LLCI = lower level of confidence interval; ULCI = upper level of confidence interval*.

**Table 5:** Hayes’ Process Regression Matrix for Moderating effect of the COP on the relationship between SOC and WE (*n* = 200).

Variables	*B (SE_s_)*	*t*	*P*	95% Confidence Interval		*R*	*R^2^*
				LLCI	ULCI		
Constant	−8.19 (3.87)	−2.11	0.04	−15.82	−0.55	0.25	0.06
SOC	2.64 (0.85)	3.10	0.02	0.96	4.31	.	
COP	4.30 (−0.89)	3.21	0.00	1.66	6.94		
Interaction_1	−0.89 (0.29)	−3.03	0.02	−1.46	−0.31		

Note: *B* = Unstandardized coefficients; SEs = standard errors *LLCI = lower level of confidence interval; ULCI = upper level of confidence interval*.
